# Sources of resistance to *Pseudocercospora fijiensis*, the cause of black Sigatoka in banana

**DOI:** 10.1111/ppa.13408

**Published:** 2021-06-12

**Authors:** Janet Kimunye, Evans Were, Rony Swennen, Altus Viljoen, George Mahuku

**Affiliations:** ^1^ International Institute of Tropical Agriculture Kampala Uganda; ^2^ Department of Plant Pathology Private Bag X1 Stellenbosch University Matieland South Africa; ^3^ Institute of Agricultural Sciences in the Tropics (Hans‐Ruthenberg‐Institute University of Hohenheim Stuttgart Germany; ^4^ Laboratory of Tropical Crop Improvement KU Leuven Leuven Belgium; ^5^ International Institute of Tropical Agriculture Arusha Tanzania; ^6^ International Institute of Tropical Agriculture Dar es Salaam Tanzania

**Keywords:** black Sigatoka, disease evaluation, resistance

## Abstract

Black Sigatoka, caused by *Pseudocercospora fijiensis*, is one of the most devastating diseases of banana. In commercial banana‐growing systems, black Sigatoka is primarily managed by fungicides. This mode of disease management is not feasible for resource‐limited smallholder farmers. Therefore, bananas resistant to *P*. *fijiensis* provide a practical solution for managing the disease, especially under smallholder farming systems. Most banana and plantain hybrids with resistance to *P*. *fijiensis* were developed using few sources of resistance, which include Calcutta 4 and Pisang Lilin. To broaden the pool of resistance sources to *P*. *fijiensis*, 95 banana accessions were evaluated under field conditions in Sendusu, Uganda. Eleven accessions were resistant to *P*. *fijiensis*. Black Sigatoka symptoms did not progress past Stage 2 (narrow brown streaks) in the diploid accessions Pahang (AA), Pisang KRA (AA), Malaccensis 0074 (AA), Long Tavoy (AA), M.A. Truncata (AA), Tani (BB), and Balbisiana (BB), a response similar to the resistant control Calcutta 4. These accessions are potential sources of *P*. *fijiensis* resistance and banana breeding programmes can use them to broaden the genetic base for resistance to *P*. *fijiensis*.

## Graphical abstract

Eleven banana accessions where symptom progression stopped at stage 2 of symptom progression were identified as potential sources of black Sigatoka resistance that can be used to broaden the genetic base for resistance to *P*. *fijiensis*.

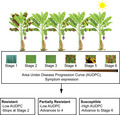

## 1 INTRODUCTION

Bananas are perennial monocot herbs belonging to the genus *Musa*, family Musaceae and order Zingiberales (Simmonds & Shepherd, 1955). The genus *Musa* comprises five sections that are divided into 40 species. Eumusa is the largest and best characterized section and includes two seeded species, *Musa acuminata* and *M*. *balbisiana*, which are the ancestors to most edible banana cultivars (Simmonds & Shepherd, 1955). Some varieties are also believed to have arisen from the hybridization of *M*. *schizocarpa* (S genome) with either *M*. *acuminata* or *M*. *balbisiana*. Recent studies revealed evidence of diversification within wild *M*. *acuminata* subspecies and intraspecific hybridization within the *M*. *acuminata* subspecies *malaccensis* and *burmannica* (Rouard et al., 2018). Southeast Asia and Indochina are the centres of diversity for banana and the region where bananas originated.

Banana is an important crop in the tropics and subtropics, and is a major staple and source of income to millions of people (FAOSTAT, 2016). Despite their importance, yields in small‐scale production systems are often low due to abiotic and biotic stresses. One of the most destructive biotic stresses of banana is black Sigatoka, a fungal leaf disease caused by *Pseudocercospora fijiensis* (Churchill, 2011). The fungus is heterothallic and produces asexual conidia and sexual ascospores throughout the year (Fouré, 1987). The disease is polycyclic and results in multiple infections in a banana cycle, leading to substantial leaf damage and yield losses of >50% (Guzmán et al., 2019). In large‐scale plantations, black Sigatoka is managed by the frequent application of fungicides (Churchill, 2011). Small‐scale farmers have limited access to fungicides and often cannot afford them (Alakonya et al., 2018). They therefore suffer massive losses from this disease.

Several banana breeding programmes have successfully developed black Sigatoka‐resistant hybrids and cultivars (Batte et al., 2019; Ortiz & Swennen, 2014; Rowe & Rosales, 2000; Tenkouano et al., 2011; Vuylsteke et al., 1993). These include the International Institute of Tropical Agriculture (IITA), where African cooking banana and plantain hybrids resistant to black Sigatoka were developed (Pillay et al., 2012; Vuylsteke et al., 1993, 1997). The plantains developed by IITA include PITA 14, PITA 21, and PITA 23, and a cooking banana hybrid called BITA 3. These hybrids are currently being grown by farmers in Cameroon, Ghana, Ivory Coast, Nigeria, and Uganda (Tenkouano et al., 2011; Tenkouano & Swennen, 2004).

In East Africa, IITA and the National Agricultural Research Organisation in Uganda (NARO) developed 27 improved East African Highland banana (EAHB) hybrids, known as NARITAs. The NARITAs have high yields, and some of them are resistant to black Sigatoka (Tushemereirwe et al., 2015). One of these hybrids, NARITA 7, has been deployed to farmers in Uganda (Nowakunda et al., 2015). The Fundacion Hondureña de Investigación Agrícola (FHIA) in Honduras has also developed improved diploids and hybrids with resistance to black Sigatoka (Pillay et al., 2012; Rowe & Rosales, 2000). The hybrids include FHIA‐17, FHIA‐18, FHIA‐21, FHIA‐23, and FHIA‐25, which are now grown in many African countries, including Ghana, Kenya, Nigeria, Tanzania, and Uganda (Tenkouano & Swennen, 2004).

The success of resistance breeding is dependent on the availability of good sources of resistance (Pillay et al., 2012). Several banana varieties resistant to black Sigatoka have been identified and used in banana improvement programmes (Pillay et al., 2012; Vuylsteke et al., 1993). Among these, Calcutta 4 (*M*. *acuminata* subsp. *burmannicoides*) and Pisang Lilin (*M*. *acuminata* subsp. *malaccensis*) are the most extensively used (Pillay et al., 2012; Vuylsteke et al., 1997). However, a vast genetic diversity does exist in bananas that may serve as potential donors of resistance (Christelová et al., 2017), but these have not been used by breeding programmes, mainly because of sterility of some of the clones and low seed set (Ortiz & Swennen, 2014).

An overreliance on a few sources of disease resistance to *P*. *fijiensis* poses a risk to the sustainability and durability of host resistance. *P*. *fijiensis* undergoes regular sexual recombination, which suggests that the fungus might overcome existing sources of resistance (McDonald & Linde, 2002). Examples of this have already been reported. Fullerton and Olsen (1995) reported that *P*. *fijiensis* isolates in Papua New Guinea and the Pacific Islands overcame resistance in young Calcutta 4 plants. In the Cook Islands, the resistant cultivars Paka and T8 (a Paka × Highgate AAAA hybrid) were reported to have become susceptible (Fullerton & Olsen, 1995). Yangambi KM5, a variety once considered highly resistant to *P*. *fijiensis* (Fouré, 1987), also became susceptible to black Sigatoka in Cameroon (Mouliom‐Pefoura, 1999), Costa Rica (Escobar‐Tovar et al., 2015), and Tanzania (Kimunye et al., 2019). In Cuba, the resistant FHIA‐18 hybrid became susceptible to *P*. *fijiensis* (Miranda et al., 2006). All these reports point to a changing pathogen virulence profile and the risk of relying on a narrow genetic pool. The existing resistant banana gene pool therefore needs to be broadened to ensure that durable resistance to black Sigatoka is being developed by *Musa* breeding programmes. The identification and introgression of new and effective *P*. *fijiensis* resistance genes into banana hybrids and cultivars has now become necessary.

Bananas and plantains have been screened for resistance to black Sigatoka before. Fouré (1994) evaluated more than 350 accessions for response to black Sigatoka in Njombe in Cameroon. However, these accessions have not been evaluated in other locations in Africa, especially in the East African highlands. Host response to infection can also depend on plantation management, including soil fertility regimes and nutrients (Kablan et al., 2012), as well as pathogen characteristics. Isolates with differing levels of aggressiveness and virulence have been reported. For example, Romero and Sutton (1997) reported higher black Sigatoka severities on Grand Naine and False Horn with isolates from Colombia, Costa Rica, and Honduras compared to those from Cameroon and Asia, while Fullerton and Olsen (1995) reported *P*. *fijiensis* strains with differential virulence from those collected in Papua New Guinea and the Pacific Islands. Banana genotypes used as resistance sources must therefore be evaluated in different environments before being used in breeding programmes.

Several accessions used by IITA and NARO in their banana breeding programmes have not been evaluated for resistance to black Sigatoka in East Africa. The objective of this study was to evaluate 95 accessions for response to *P*. *fijiensis* in Uganda (under highland conditions), including 13 accessions previously evaluated in Cameroon (under lowland conditions), along with wild and improved diploids. This was done to identify additional sources of resistance that could potentially be used as parents in IITA and NARO’s banana breeding programmes.

## 2 MATERIALS AND METHODS

### 2.1 Plant materials and trial design

Two trials were conducted to test banana accessions in the IITA germplasm collection for resistance against *P*. *fijiensis* under natural field conditions (natural infection) at Sendusu, Wakiso district in Uganda. The station lies at 0.53°N, 32.58°E, 1,150 m a.s.l. Rainfall is about 1200 mm/year, with a bimodal distribution between two rainy seasons, March–June and September–December. The annual minimum temperature is 17.9 ℃, maximum temperature 29.1 ℃, average temperature 22 ℃, and a relative humidity of 76.3%. These conditions are favourable for *P*. *fijiensis* infection and proliferation.

Trial 1 comprised 79 diverse *Musa* accessions originating from different geographic regions (Table 1). The trial was planted in 2013 using tissue culture plantlets. The accessions were planted as unreplicated single row plots, with five plants per row at a spacing of 3 × 3 m. From each of the five mats, one fully developed sucker with foliage (maiden sucker) per mat was selected, tagged, and evaluated every 3 months in 2017 until the plants were harvested. Three evaluations were made per plant. Calcutta 4 and Mbwazirume plants were used as resistant and susceptible checks, respectively.

**TABLE 1:** Banana accessions from the germplasm collection (Trial 1) maintained at the IITA banana research farm in Sendusu, Uganda, and their response to infection by *Pseudocercospora fijiensis* under field conditions

ITC number	Accession	Ploidy^a^	Species/subgroup^b^	Cluster^b^	Black Sigatoka evaluation parameter						Reaction type^d^
					AUDPC	SSD	YLst	YLS^c^	INSL	DSI	
ITC0249	Calcutta 4^e^, ^f^	AA	*Musa acuminata* subsp. *burmannica*	I	36.0 a	2	8.3	10.4	100.0	3.9	Resistant
ITC0213	Pisang Awak	ABB	Pisang Awak	VIII	39.1 ab	2	8.8	10.9	100.0	6.0	Resistant
ITC0609	Pahang^f^	AA	*M*. *acuminata* subsp. *malaccensis*	III	55.9 a–c	2	7.5	10.8	100.0	7.2	Resistant
ITC1345	Pisang KRA	AA	*M*. *acuminata* subsp. *malaccensis*	III	71.2 a–d	2	6.5	9.4	100.0	10.3	Resistant
ITC1143	Giahui	ABB	Pisang Awak	I	81.1 a–e	2	7.7	10.1	100.0	2.5	Resistant
ITC0074	Malaccensis	AA	*M*. *acuminata* subsp. *malaccensis*	III	73.7 a–e	2	5.9	8.5	100.0	8.1	Resistant
ITC1120	Tani	BB	*Musa balbisiana*	VII	92.5 a–f	2	8.6	14.5	100.0	12.9	Resistant
MMC192	Balbisiana	BB	*M. balbisiana*	VII	110.3 a–h	2	6.2	13.6	100.0	16.0	Resistant
ITC0393	M.A. Truncata	AA	*M*. *acuminata* subsp. *truncata*	I	144.7 d–k	2	4.1	7.7	100.0	7.2	Resistant
ITC1349	Pisang Serun 400	AA	*M*. *acuminata* subsp. *malaccensis*	III	41.6 ab	3	8.1	10.8	100.0	12.2	Resistant
ITC0246	Cameroun	BB	*M. balbisiana*	VII	43.3 ab	3	8.5	12.6	100.0	17.1	Resistant
ITC1348	Pisang Serun 404	AA	*M*. *acuminata* subsp. *malaccensis*	III	73.6 a–e	3	6.4	9.6	100.0	12.9	Resistant
ITC0250	Malaccensis	AA	*M*. *acuminata* subsp. *malaccensis*	III	80.1 a–e	3	6.7	10.5	100.0	10.8	Resistant
ITC1139	Zebrina	AA	*M*. *acuminata* subsp. *zebrina*	X	82.6 a–e	3	6.4	9.5	100.0	8.4	Resistant
ITC1177	Zebrina	AA	*M*. *acuminata* subsp. *zebrina*	X	90.0 a–e	3	5.8	8.3	100.0	10.2	Resistant
ITC0610	Tuu Gia^f^	AA	Unknown	I	94.0 a–f	3	7.1	10.8	100.0	9.3	Resistant
ITC0728	Maia Oa	AA	*M*. *acuminata* subsp. *zebrina*	X	103.9 a–h	3	5.6	8.0	100.0	9.4	Resistant
ITC0944	Wambo	AA	Unknown	XIII	111.0 a–i	3	7.1	9.5	100.0	10.1	Resistant
ITC0526	K.N. Khom	ABB	Pisang Awak	VIII	115.4 b–i	3	6.0	11.3	100.0	18.1	Resistant
ITC1179	Monyet	AA	*M*. *acuminata* subsp. *zebrina*	X	112.7 a–i	3	5.1	8.3	100.0	17.7	Resistant
ITC0087	Kayinja	ABB	Pisang Awak	VIII	123.9 c–j	3	6.5	12.0	100.0	19.4	Resistant
ITC1121	Pisang Lilin^f^	AA	ISEA 1	III	126.6 c–j	3	6.2	9.4	100.0	15.8	Resistant
MMC166	Kisubi	AB	Ney Poovan	VIII	150.9 e–l	3	5.9	10.8	100.0	21.7	Resistant
ITC1000	Gunih	AA	Unknown		231.7 m–r	3	5.6	10.0	100.0	2.5	Resistant
ITC0966	Zebrina GF	AA	*M*. *acuminata* subsp. *zebrina*	X	229.0 l–q	4	7.3	13.1	100.0	20.5	Resistant
ITC1441	Pisang Ceylan^f^	AAB	Mysore	VIII	232.8 m–r	4	6.8	11.1	100.0	23.7	Resistant
ITC1319	FHIA 18	AAAB	Pome	IX	162.0 f–m	4	6.3	10.3	100.0	17.4	Resistant
ITC0058	Cacambou^f^	ABB	Bluggoe/Monthan	XII	162.2 f–m	4	6.1	8.5	100.0	23.3	Resistant
ITC0947	Duningi	AAB	Indon TriPri	X	63.5 a–c	6	4.0	6.2	65.2	21.9	Intermediate
ITC0814	Bagul	AA	*M*. *acuminata* subsp. *banksii*	XI	79.6 a–e	6	4.6	7.6	88.3	9.9	Intermediate
ITC1243	Kokopo	AA	*M*. *acuminata* subsp. *malaccensis*	III	98.9 a–g	6	5.5	8.5	73.6	15.8	Intermediate
ITC1178	Buitenzorg	AA	ISEA 2	III	106.5 a–h	6	5.5	6.8	88.2	35.5	Intermediate
MMC001	Namadhi	AAA	Lujugira/Mutika	X	132.9 c–j	6	4.0	4.6	61.7	32.3	Intermediate
ITC0712	Cultivar Rose	AA	*M*. *acuminata* subsp. *malaccensis*	III	143.0 d–k	6	4.9	7.6	76.5	14.1	Intermediate
ITC0310	Morong Princesa	AA	Pisang Jari Buaya	I	143.3 d–k	6	6.4	10.1	80.9	17.8	Intermediate
ITC0837	Yalim	AA	*M*. *acuminata* subsp. *zebrina*	X	180.4 h–o	6	4.9	7.3	69.9	22.0	Susceptible
ITC0629	Selangor	AA	Related to AA cv. African	IX	193.6 i–o	6	6.4	8.9	78.8	29.5	Susceptible
ITC0010	0010 Bluggoe	ABB	Bluggoe/Monthan	XII	194.9 j–o	6	3.3	5.3	78.2	36.8	Susceptible
ITC0364	Silver Bluggoe	ABB	Bluggoe/Monthan	XII	203.0 j–p	6	6.0	8.5	78.6	22.4	Susceptible
ITC0019	I.C.2^f^	AAAA	Related to AA African cultivar	IX	207.7 j–p	6	5.8	8.8	78.7	27.1	Susceptible
ITC1318	SH 3436‐9	AAAA	Related to AA African cultivar	IX	208.1 j–p	6	5.0	7.5	63.1	25.5	Susceptible
ITC0840	Kuspaka	AA	*M*. *acuminata* subsp. *banksii*	XI	218.6 k–p	6	4.4	7.1	68.6	20.2	Susceptible
ITC0868	Pora Pora	AA	*M*. *acuminata* subsp. *banksii*	XI	219.8 k–p	6	4.6	8.4	78.3	29.6	Susceptible
ITC1467	Kisanga Machi	AA	ISEA 2	IX	222.0 k–p	6	6.2	9.5	80.4	29.6	Susceptible
ITC0116	Saba^f^	ABB	Bluggoe/Monthan	XII	225.0 l–p	6	6.0	9.3	83.5	26.2	Susceptible
ITC0595	Pagatau	AAA	Indon TriNG	IX	225.6 l–q	6	6.1	8.3	71.1	33.7	Susceptible
ITC0396	Pelipita^f^	ABB	Pelipita (*M*. *balbisina* cluster)	VII	234.7 m–s	6	7.1	9.7	69.7	24.3	Susceptible
ITC1305	Paji	AA	Lujugira/Mutika	X	235.8 m–s	6	6.3	9.3	67.0	24.0	Susceptible
ITC0768	Lacatan^f^	AAA	Cavendish	IX	236.0 m–s	6	5.2	7.3	74.0	27.6	Susceptible
ITC1464	Ntindi II	AAA	Lujugira/Mutika	X	239.4 m–s	6	6.4	9.6	77.3	30.0	Susceptible
ITC0084	Mbwazirume^f^, ^g^	AAA	Lujugira/Mutika	X	245.4 n–t	6	4.4	5.6	65.3	32.1	Susceptible
MMC167	Sukari Ndizi	AAB	Karamasenge (Silk cluster)	VIII	247.1 n–t	6	5.7	8.9	80.4	22.0	Susceptible
ITC1466	Nshonowa	AA	AA African cultivar	IX	254.0 o–t	6	3.8	5.5	60.3	35.2	Susceptible
TARS18062	Pitu	AA	AA African cultivar	IX	259.1 o–t	6	5.1	7.1	65.8	29.6	Susceptible
ITC1458	Ilayi Red	AAA	Lujugira/Mutika	X	261.1 o–t	6	4.1	5.9	63.0	29.0	Susceptible
ITC0897	Banksii 897	AA	*M*. *acuminata* subsp. *banksii*	XI	263.3 o–t	6	5.5	8.0	76.4	21.6	Susceptible
ITC1461	Ntebwa	AAA	Lujugira/Mutika	X	263.9 o–t	6	6.1	9.5	75.2	34.9	Susceptible
ITC1594	Mshale	AA	AA African cultivar	IX	267.2 o–t	6	6.6	8.0	67.2	36.1	Susceptible
ITC1224	Kikundi	AAA	Lujugira/Mutika	X	280.0 p–v	6	6.9	9.3	72.1	31.8	Susceptible
ITC0243	Pisang Radjah	AAB	Nendra Padaththi (Pome cluster)	IX	281.0 p–v	6	6.3	7.5	61.4	33.7	Susceptible
ITC0312	Pisang Jari Buaya	AA	Pisang Jari Buaya	I	282.1 p–v	6	4.8	6.5	62.0	26.4	Susceptible
ITC1544	Mlelembo	AA	AA African cultivar	IX	282.3 p–v	6	5.4	7.4	76.7	39.3	Susceptible
ITC0654	Petite Naine	AAA	Cavendish	IX	282.8 p–v	6	3.9	5.6	57.2	30.7	Susceptible
ITC1454	Makyugu 1	AA	AA African cultivar	IX	286.9 p–v	6	5.5	6.9	63.8	30.6	Susceptible
ITC1457	Haa Haa	AAA	Lujugira/Mutika	X	303.1 q–v	6	4.9	7.5	68.1	22.6	Susceptible
ITC0946	Merik	AAA	Indon TriNG	IX	304.1 q–v	6	4.1	6.2	53.0	31.7	Susceptible
ITC0259	Galeo	AA	AA African cultivar	IX	309.0 r–v	6	4.7	8.0	58.2	29.5	Susceptible
MMC016	Tereza	AAA	Lujugira/Mutika	X	314.0 s–w	6	4.0	6.4	71.6	22.0	Susceptible
ITC1462	Suu	AAA	Lujugira/Mutika	X	317.0 s–w	6	4.2	6.5	60.0	23.0	Susceptible
ITC0164	0164 Rugondo	AAA	Lujugira/Mutika	X	323.9 s–x	6	4.1	6.5	69.3	28.4	Susceptible
ITC1452	Huti Shumba	AA	AA African cultivar	IX	324.7 t–x	6	4.7	6.5	58.6	38.1	Susceptible
ITC1465	Ibwi	AAA	AA African cultivar	IX	356.8 u–x	6	4.0	7.1	75.3	26.6	Susceptible
ITC1456	Huti RB	AA	AA African cultivar	IX	359.1 v–x	6	4.4	6.3	54.7	45.0	Susceptible
MMC020	Kibuzi	AAA	Lujugira/Mutika	X	388.2 v–x	6	4.1	5.6	59.1	36.5	Susceptible
ITC0574	Robusta^f^	AAA	Cavendish	IX	389.2 wx	6	4.5	6.4	53.0	38.0	Susceptible
ITC1451	Kitarasha	AAA	Lujugira/Mutika	X	392.3 wx	6	5.5	7.6	65.9	21.7	Susceptible
ITC0078	Whogu	AAA	Indon TriNG	IX	401.2 x	6	4.9	6.9	68.7	30.3	Susceptible
ITC1468	Kahuti	AA	AA African cultivar	IX	414.4 x	6	4.4	6.4	59.3	40.7	Susceptible
ITC1459	Mlema	AAA	Lujugira/Mutika	X	426.9 x	6	4.7	7.4	62.5	34.9	Susceptible
LSD							1.1	1.4	11.1	10.7	

Note

Disease parameters collected at 3‐month intervals and averaged over one crop cycle.

Abbreviations: AUDPC, area under disease progress curve; YLst, youngest leaf with streak symptoms; YLS, youngest leaf spotted; SSD, most advanced stage of symptoms; INSL index of nonspotted leaves (%); DSI, disease severity index.

^a^ Genome group and ploidy level assignment was based on *Musa* Germplasm Information System.

^b^ Accessions grouped together based on the morphological traits and into cluster as defined using simple sequence repeats (Christelová et al., 2017; Nakato et al., 2018).

^c^ YLS value on accessions without Stage 6 lesions is number of standing leaves plus 1 (NSL+1).

^d^ Reaction type assigned based on hierarchical clustering using AUDPC, SSD, YLst, YLS, INSL, and DSI.

^e^ Calcutta 4 resistant check with high resistance (Carlier et al., 2003).

^f^ Cultivar previously evaluated at Njombe, Cameroon (Fouré, 1994; Guzmán et al., 2019).

^g^ Mbwazirume: an East African Highland banana used as a susceptible local check (Tushemereirwe et al., 2015).

Trial 2 was planted in 2017 and included 22 accessions (Table 2). Eight of these were selected from the first trial based on their response to *P*. *fijiensis*, five were diploid accessions previously used to generate improved diploids, six were improved diploids, and three were tetraploids used in the NARO/IITA breeding pipeline. Yangambi KM5 was included to validate the reduced resistance observed in farmers’ fields (Kimunye et al., 2019), while Williams and Mbwazirume served as susceptible checks, and Calcutta 4 as resistant check.

**TABLE 2:** The response of banana accessions including diploids, triploids, and tetraploids (Trial 2) to infection by *Pseudocercospora fijiensis*. Symptoms under field conditions in Uganda were scored based on the area under disease progress curve (AUDPC) and the most advanced stage of disease symptom

ITC number	Accession name	Ploidy^a^	Species^b^	Cluster^b^	Black Sigatoka assessment parameters						Reaction type^d^
					AUDPC	SSD	YLst	YLS^c^	INSL	DSI	
ITC0093	Long Tavoy	AA	*Musa acuminata* subsp. *burmannica*	I	39.8 a	2	7.9	11.0	100.0	5.7	Resistant
ITC0249	Calcutta 4^e^	AA	*M*. *acuminata* subsp. *burmannica*	I	57.0 ab	2	6.5	9.7	100.0	7.0	Resistant
ITC0074	Malaccensis	AA	*M*. *acuminata* subsp. *malaccensis*	III	68.1 ab	2	5.9	9.5	100.0	9.2	Resistant
ITC0609	Pahang	AA	*M*. *acuminata* subsp. *malaccensis*	III	60.2 ab	3	6.6	10.9	100.0	7.7	Resistant
ITC0253	Borneo	AA	*M*. *acuminata* subsp. *microcarpa*	X	61.7 ab	3	6.6	10.2	100.0	8.1	Resistant
ITC1121	Pisang Lilin	AA	ISEA 1	III	79.0 bc	3	5.1	8.9	100.0	11.9	Resistant
	**02145/1320**	AA			106.6 cd	3	4.6	9.8	100.0	15.9	Resistant
ITC1179	Monyet	AA	*M*. *acuminata* subsp. *zebrina*	X	68.8 ab	4	5.3	8.8	100.0	9.4	Resistant
ITC1123	Yangambi KM5^f^	AAA	Ibota	III	107.5 cd	6	5.4	10.0	90.9	13.5	Intermediate
	**10969S‐1**	AA			116.1 d–f	6	5.5	7.4	74.8	17.5	Susceptible
	**TMB2X5265‐1**	AA			116.1 d–f	6	4.9	9.1	87.2	17.8	Intermediate
ITC0966	Zebrina GF	AA	*M*. *acuminata* subsp. *zebrina*	X	126.2 d–g	6	5.6	10.8	83.4	17.8	Intermediate
	**1438k‐1**	AAAA			126.9 d–h	6	4.6	7.1	74.0	19.2	Susceptible
ITC1243	Kokopo	AA	AA cultivar		133.0 d–h	6	4.9	6.8	62.6	21.9	Susceptible
	**222K‐1**	AAAA			134.6 d–h	6	4.2	5.7	63.4	22.7	Susceptible
	**376K‐7**	AAAA			137.2 e–h	6	4.7	6.8	73.0	20.1	Susceptible
ITC1545	Mwitu Pemba	AAB	Silk	VIII	139.7 f–h	6	5.7	8.6	68.4	22.7	Susceptible
MMC214	Cultivar Rose	AA	*M*. *acuminata* subsp. *malaccensis*	III	142.8 f–h	6	4.4	6.4	67.9	20.9	Susceptible
MMC218	**SH3217**	AA	Unknown		151.3 gh	6	5.2	7.1	67.9	22.0	Susceptible
ITC059	Kasaska	AA	Unknown		159.8 hi	6	5.4	8.1	68.2	23.7	Susceptible
MMC414	**SH3362**	AA	Unknown		188.5 ij	6	5.0	7.1	64.8	25.2	Susceptible
MMC251	**TMB2X9128‐3**	AA			208.3 jk	6	4.7	6.2	58.4	29.5	Susceptible
ITC0084	Mbwazirume^g^	AAA	Lujugira/Mutika	X	214.9 jk	6	4.5	5.5	56.4	29.3	Susceptible
	Mchare Laini	AA	AA African cultivar	IX	225.1 kl	6	4.9	6.2	58.9	34.8	Susceptible
ITC0365	Williams^g^	AAA	Cavendish	IX	253.2 l	6	4.6	5.6	49.7	36.3	Susceptible
LSD							0.5	0.6	5.5	2.7	

Note

Accessions in bold are the improved diploids and tetraploids.

Abbreviations: AUDPC, area under disease progress curve; YLst, youngest leaf with streak symptoms; YLS, youngest leaf spotted; SSD, most advanced stage of symptoms; INSL index of nonspotted leaves (%); DSI, disease severity index. Disease parameters collected at 3‐month intervals and averaged over three crop cycles.

^a^ Genome group and ploidy level assignment was based on *Musa* Germplasm Information System.

^b^ Accessions grouped into clusters as defined using simple sequence repeats (Christelová et al., 2017; Nakato et al., 2018).

^c^ YLS value on accessions without Stage 6 symptoms is number of standing leaves plus 1 (NSL+1).

^d^ Reaction type assigned based on hierarchical clustering using AUDPC, SSD, YLst, YLS, INSL, and DSI.

^e^ Calcutta 4 resistant check with high resistance (Carlier et al., 2003).

^f^ Cultivar previously evaluated at Njombe, Cameroon (Fouré, 1994; Guzmán et al., 2019).

^g^ Mbwazirume, an East African Highland banana, and Williams were used as susceptible local checks.

The field experiment consisted of rows comprising seven plants per accession, of which five were used for disease ratings, planted in a randomized complete block design with three replications. The trial was established with suckers collected in Sendusu and Kawanda. Plants were planted with a spacing of 2 × 3 m. The suckers were pared before planting, and the rhizomes treated with Dursban (chlorpyrifos) for 20 min to eliminate nematodes and weevils. A *P*. *fijiensis*‐susceptible Matooke variety (EAHB, cooking type), Enzirabahima, was used as a disease spreader row to ensure there was enough inoculum in the field. The accessions were evaluated every 3 months, starting at 6 months after planting, for three crop cycles (mother plant, daughter, and granddaughter), concluding evaluations in November 2018. Each of the cycles lasted 9–12 months depending on the cultivar.

Field management of the two trials was similar. At planting each hole was filled with 10 kg cow manure, after which dry grass was applied as mulch 4 months after planting. Weeding was done by hand until flowering. A herbicide (Weedall, a glyphosate‐based nonselective herbicide) was thereafter used to manage weeds. Detrashing was minimal and limited to dry leaves hanging around the pseudostem.

### 2.2 Disease evaluation

**FIGURE 1:**
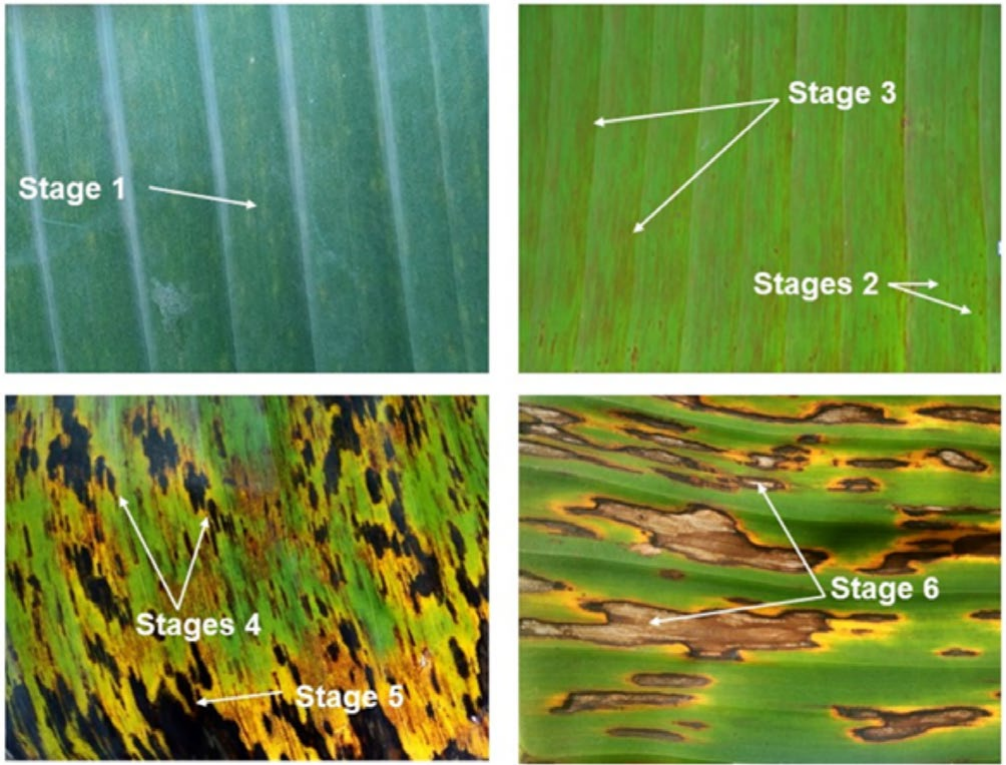
Pictorial representation of black Sigatoka symptoms [Colour figure can be viewed at wileyonlinelibrary.com]

Disease was scored by counting the number of standing leaves (NSL). Each leaf was visually rated for the stage of symptom development, as described by Fouré (1987): Stage 1, development of faint, minute, reddish‐brown specks on the lower surface of the leaf; Stage 2, narrow reddish‐brown streaks; Stage 3, streaks that change colour from reddish‐brown to dark brown or black that are clearly visible at the upper surface of the leaf; Stage 4, streaks broaden and become spindle‐shaped with water‐soaked borders; Stage 5, lesions with dark brown or black centres that are slightly depressed with water‐soaked borders; and Stage 6, grey lesions with dried out centres (Figure 1).

Disease severity was evaluated on a 0–6 scale (Gauhl, 1994). According to this scale, 0 = no visible symptoms, 1 = <1%, 2 = 1%–5%, 3 = 6%–15%, 4 = 16%–33%, 5 = 34%–50%, and 6 = 51%–100% of leaf area covered with disease symptoms. At the most advanced stage of symptoms (SSD), that is, the stage at which symptom progression stopped on each plant (all leaves), the youngest leaf with visible streak symptoms (Stage 2; YLst) and the youngest leaf spotted (YLS) were recorded.

The index of nonspotted leaves (INSL) was computed as (YLS − 1)/NSL × 100. Disease severity scores per leaf were used to compute the disease severity index per plant:

$$\mathrm{DSI}=\frac{\sum nb}{\left(N‐1\right)T}\;\times 100$$

where *n* = number of leaves in each severity grade, *b* = grade (0–6), *N* = number of severity grades used in the scale (7), and *T* = total number of leaves scored.

Disease severity index over the different evaluation times was used to calculate the area under disease progress curve (AUDPC), using the formula:

$$\text{AUDPC}=\sum n_{i}=1\left[\left(X_{i}+1+X_{i}\right)/2\right]\;\left[t_{i}+1‐t_{i}\right]$$

where *X_i_* = proportion of the host tissue damaged at *i*th day, *t_i_* = the time in months after appearance of the disease at *i*th month, and *n* = the total number of observations.

### 2.3 Data analysis

Variation among accessions was assessed using one‐way analysis of variance (ANOVA), and the means separated using the least significant difference at the 95% confidence level. For the second trial, no significant differences between the mother, daughter, and granddaughter plants were obtained, so the data were combined and subjected to an ANOVA. Pearson's correlation was used to determine the association between the different disease parameters, AUDPC, SSD, INSL, YLS, and YLst. Mean values from the ANOVA were used to perform a cluster analysis based on Euclidean distances. Hierarchical clustering of banana accessions was done using AUDPC, INSL, YLS, YLst, and SSD. All the analysis was implemented in GenStat v. 19 (VSN International Ltd).

### 2.4 Genetic grouping

Accessions were assigned to different genomic groups and ploidy level according to the *Musa* Germplasm Information system (MGIS) database (https://www.crop‐diversity.org/mgis/). The accessions were then grouped into genetic clusters based on simple‐sequence repeat (SSR) genotyping data (Christelová et al., 2017; Nakato et al., 2018).

## 3 RESULTS

### 3.1 Black Sigatoka symptoms

Black Sigatoka symptoms were observed on all banana accessions evaluated. The symptoms ranged from Stage 2 to the late necrotic stage (Stage 6) (Figure 1). In some accessions such as Calcutta 4, Pisang Awak, Pahang, Pisang KRA, Giahui, Malaccensis 0074, Tani, Balbisiana and M.A. Truncata (Trial 1, Table 1), as well as Long Tavoy, Calcutta 4, and Malaccensis 0074 (Trial 2, Table 2), symptoms did not progress beyond Stage 2. In 19 accessions, symptom progression stopped at Stage 3, including in Pisang Serun, Cameroun, Malaccensis 250, Zebrina 1177, Zebrina 1139, and Pisang Lilin (Trial 1, Table 1), as well as in Pahang, Borneo, Pisang Lilin, and 02145/1320 (Trial 2, Table 2). In some entries, including Zebrina GF, Pisang Ceylan, FHIA 18, and Cacambou in Trial 1, and Monyet in Trial 2, symptoms developed to Stage 4, but did not progress to the late necrotic stage. Black Sigatoka symptoms on the rest of the accessions progressed to Stage 6 (Tables 1 and 2).

### 3.2 Relationship between disease parameters

Significant correlations (*p* < 0.0001) were observed between black Sigatoka assessment parameters in both screening trials (Table 3). YLS and INSL were positively correlated to YLst in Trial 1 (*r* = 0.85 and *r* = 0.64) and Trial 2 (*r* = 0.77 and *r* = 0.67), respectively, and DSI and AUDPC were positively correlated in Trial 1 (*r* = 0.82) and Trial 2 (*r* = 0.97), respectively. INSL was negatively correlated to SSD in the two trials (*r* = −0.87 and *r* = −0.81). DSI and YLst had the lowest correlation both in Trial 1 (*r* = −0.33) and Trial 2 (*r* = −0.48). For both trials, the most advanced SSD had the highest coefficient of determination (*R* ^2^ = 0.77 in Trial 1 and *R* ^2^ = 0.89 in Trial 2) (Table 3). SSD and AUDPC had a higher coefficient of determination than DSI, YLS, YLst, and INSL for the two trials, and were therefore used in subsequent analysis (Table 3).

**TABLE 3:** Pearson coatoka evaluation parameters

Trial	Variable	DSI	INSL	YLS	YLst	SSD	AUDPC	*R* ^2^
1	DSI	1						0.26
	INSL	−0.84	1					0.46
	YLS	−0.63	0.75	1				0.44
	YLst	−0.33	0.64	0.85	1			0.22
	SSD	0.63	−0.87	−0.68	−0.62	1		0.77
	AUDPC	0.82	−0.77	−0.55	−0.54	0.71	1	0.69
2	DSI	1						0.36
	INSL	−0.95	1					0.44
	YLS	−0.82	0.89	1				0.35
	YLst	−0.48	0.67	0.77	1			0.17
	SSD	0.78	−0.81	−0.80	−0.69	1		0.89
	AUDPC	0.97	−0.93	−0.68	−0.67	0.75	1	0.64

Abbreviations: DSI, disease severity index; INSL, index of nonspotted leaves; YLS, youngest leaf spotted; YLst, youngest leaf with streak symptoms; SSD, overall most advanced stage of symptoms observed; AUDPC, area under disease progress curve.

### 3.3 Genotype response to black Sigatoka

#### 3.3.1 Trial 1

Banana accessions responded differently (*p* < 0.05) to black Sigatoka (Table 1). Calcutta 4, used as a resistant check, had the lowest disease severity, with an AUDPC of 36.0, while the most susceptible accession was Mlema (AUDPC = 426.9) (Table 1). Disease severity in several accessions did not differ significantly from Calcutta 4, and these were classified as resistant (Table 1). Other than Calcutta 4, the resistant check, other highly resistant accessions with an AA genome were Pahang, Pisang KRA, Malaccensis 0074, and M.A. Truncata. The other highly resistant accessions were Tani and Balbisiana within the BB genome group (Table 1).

**FIGURE 2:**
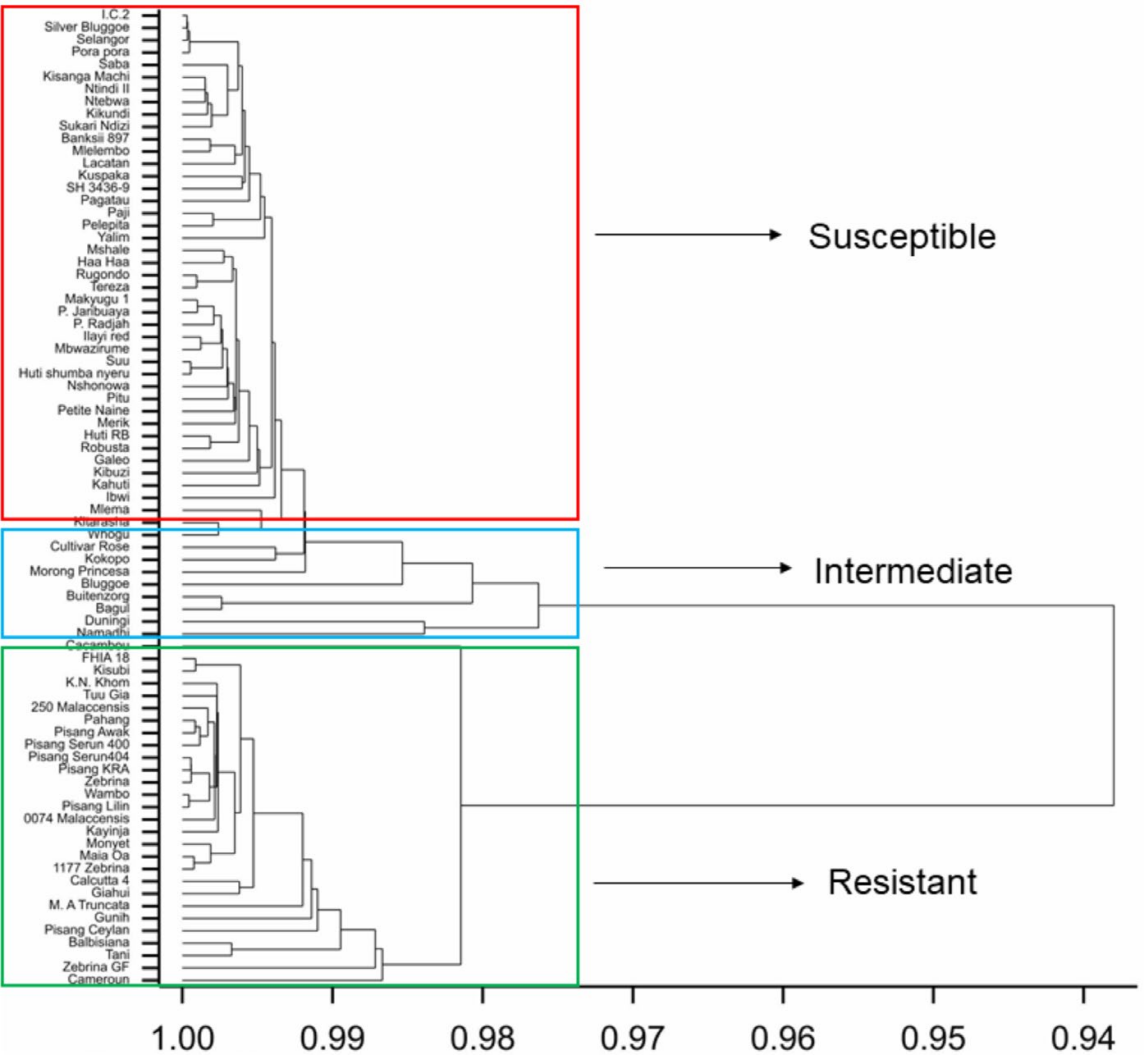
Dendrogram of a hierarchical cluster analysis for the response of banana accessions in the IITA germplasm collection (Trial 1) at Sendusu, Uganda, when evaluated for resistance against *Pseudocercospora fijiensis* under field conditions. Clustering is based on the Euclidean distances for area under disease progress curve, index of nonspotted leaves, youngest leaf spotted, the youngest leaf with streak symptoms, and the stage of most advanced symptoms [Colour figure can be viewed at wileyonlinelibrary.com]

Hierarchical clustering revealed three groups representing resistant and susceptible accessions, while some accessions had an intermediate response. The resistant group comprised 28 accessions that clustered with Calcutta 4 (Figure 2). Some of the accessions in this group, such as K.N. Khom, Kayinja, and Pisang Lilin, had a significantly higher AUDPC than Calcutta 4, but with symptoms that did not progress beyond Stage 4 (Table 1). The second group consisted of 43 accessions that clustered with the susceptible accession Mbwazirume, while eight accessions were considered intermediate (Figure 2). The intermediate accessions had a significantly lower AUDPC than Mbwazirume, but with symptoms progressing to Stage 6 (Table 1).

#### 3.3.2 Trial 2

**FIGURE 3:**
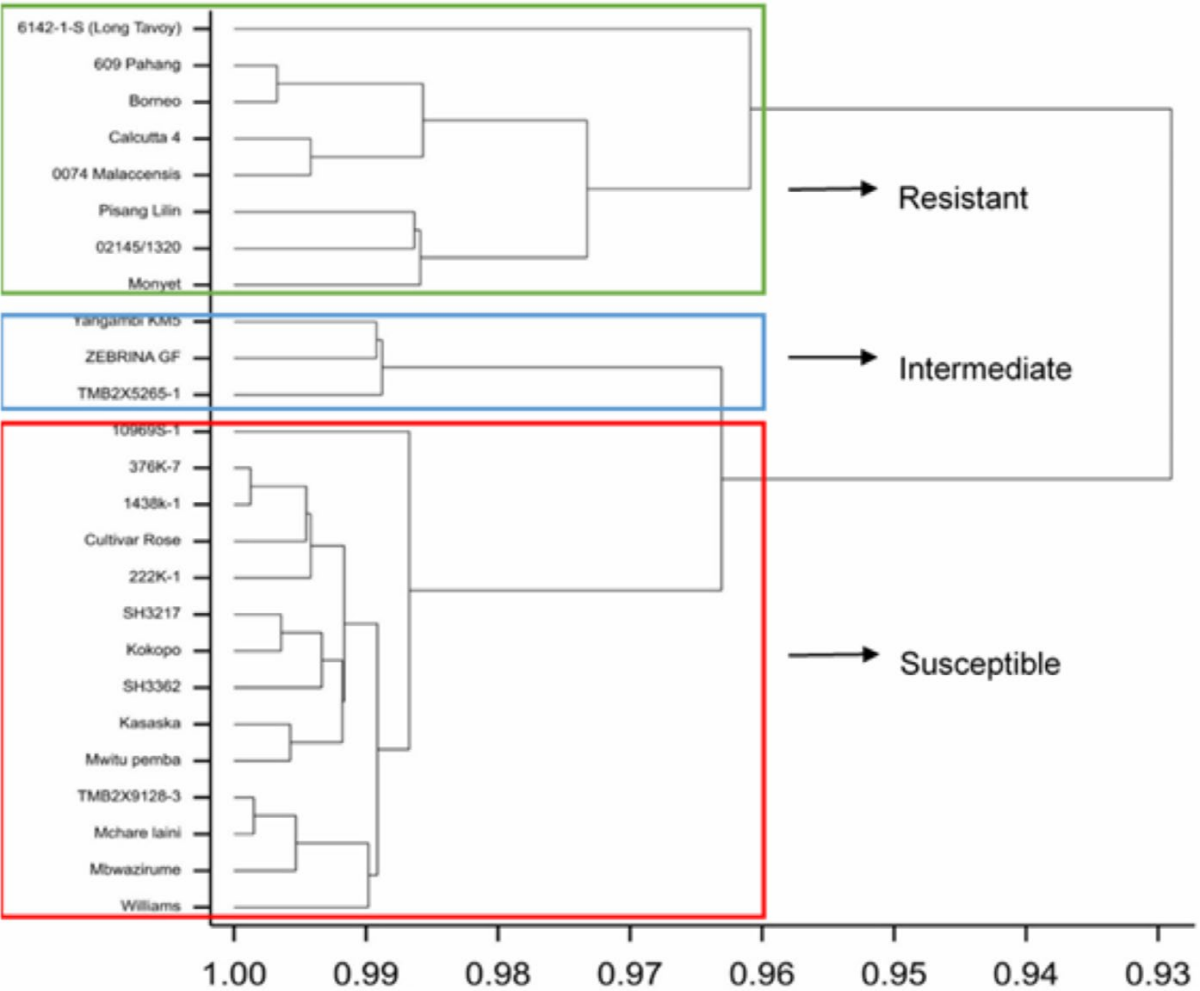
Dendogram of hierarchical cluster analysis for the response of selected banana accessions and breeding materials (Trial 2) evaluated against *Pseudocercospora fijiensis* under field conditions at Sendusu, Uganda. Clustering is based on Euclidean distances for area under disease progress curve, index of nonspotted leaves, youngest leaf spotted, the youngest leaf with streak symptoms, and the stage of most advanced symptoms [Colour figure can be viewed at wileyonlinelibrary.com]

The response of the banana accessions to *P*. *fijiensis* in Trial 2 varied significantly (*p* < 0.05) (Table 2). The accessions also grouped into three clusters. Long Tavoy, followed by Calcutta 4, were most resistant, with AUDPC values of 39.8 and 57.0, respectively (Table 2). Other accessions that clustered with Calcutta 4 and Long Tavoy were Pahang, Borneo, Malaccensis, Pisang Lilin, 02145/1320, and Monyet (Table 2; Figure 3). Yangambi KM5, Zebrina GF, and TMB2X5265‐1 were in the intermediate group.

All improved diploid and tetraploid bananas evaluated in this study were susceptible to *P*. *fijiensis*, except accessions 02145/1320 and TMB2X5265‐1 (Table 2). Improved diploids TMB2X9128‐3, SH 3217, SH 3362, and 10969S‐1, and the tetraploids 222k‐1,1438k‐1, and 376K‐1, were susceptible to *P*. *fijiensis* and clustered with the susceptible cultivars Williams and Mbwazirume (Figure 3).

In the two trials, 31 accessions were considered resistant to black Sigatoka, of which one was an improved diploid (02145/1320), 25 were wild diploids (21 *M*. *acuminata*, four *M*. *balbisiana*), five were triploid *M*. *balbisiana* bananas, and one was the tetraploid FHIA 18 hybrid (Tables 1 and 2).

### 3.4 Genetic grouping

**FIGURE 4:**
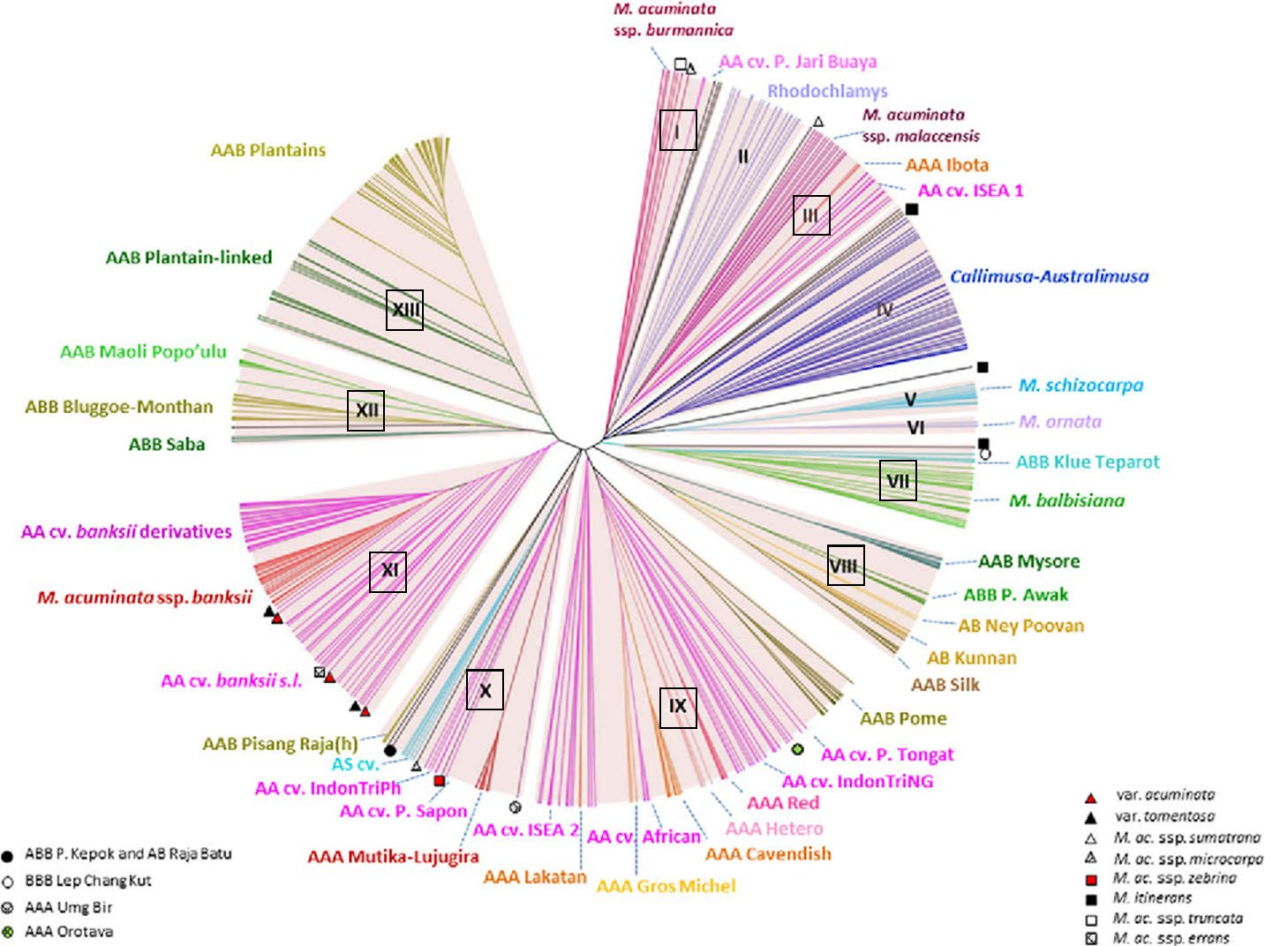
Simple‐sequence repeat (SSR)‐based genetic clusters (with a bold rectangle around them), into which the accessions assessed for response to infection with *Pseudocercospora fijiensis* were categorized. The 95 accessions grouped into Clusters I, III, VII, VIII, IX, X, XI, and XII as indicated by Christelová et al. (2017). The individual sets of the clustered accessions are indicated in Tables 1 and 2. The diagram was adopted and modified from Christelová et al. (2017) [Colour figure can be viewed at wileyonlinelibrary.com]

The accessions evaluated were distributed across 22 subgroups within nine clusters (Table 4; Figure 4). The *M*. *acuminata* group had the highest number of resistant accessions (67.7%), while 32.3% of the accessions belonged to the *M*. *balbisiana* group (Table 4). Resistant accessions were found in 12 of the subgroups, with *M*. *acuminata* subsp. *malaccensis* (III), *M*. *acuminata* subsp. *zebrina* (X), and *M*. *acuminata* subsp. *burmannica* (I) having the highest number of resistant accessions (Table 4). Most of the susceptible accessions were in the subgroup AAA Lujugira/Mutika (Cluster X) and AA cv. African (Cluster IX), with 15 and 14 accessions, respectively (Table 4).

**TABLE 4:** Groups of banana accessions with resistant and susceptible response to *Pseudocercospora fijiensis*, the cause of black Sigatoka, in Uganda

Species	Subgroup	Genetic cluster	No. of accessions		
			Resistant^a^	Intemediate^b^	Susceptible^c^
*Musa acuminata*	*burmannica*	I	4	—^d^	—
	*malaccensis*	III	6	2	
	*zebrina*	X	5	—	2
	*microcarpa*	X	1	—	—
	Unknown^e^		4	1	8
	ISEA 1	III	1	—	—
	ISEA 2	IX	—	1	1
	*banksii*	XI	—	1	3
	Ibota	III	—	1	—
	Cavendish	IX	—		4
	Pisang Jari Buaya	I	—	1	—
	Indon TriNG	IX	—	‐—	3
	Indon TriPri	X	—	1	
	AA African cultivar	IX	—	—	14
	Lujugira/Mutika	X	—	1	14
*Musa balbisiana*	*M. balbisiana*	VII	3	—	1
	Ney Poovan	VIII	1	—	—
	Mysore	VIII	1	—	—
	Pome^f^	VIII	1	—	1
	Pisang Awak	VIII	3	—	—
	Bluggoe/Monthan	XII	1	1	1
	Silk	VIII	—	—	2
Total	22	9	31	10	54

^a^ Resistant refers to accessions with low disease severity and symptom progression stopped at early streak Stage 2–4.

^b^ These accessions had moderate disease severity with symptoms progressing to late necrotic Stage 6.

^c^ These accessions had high disease severity with symptoms progressing to late necrotic Stage 6.

^d^ No accessions in the genetic block with that response to black Sigatoka.

^e^ Most of the accessions with unknown genetic group are the improved diploids and tetraploid breeding materials.

^f^ The resistant accession under Pome subgroup is FHIA 18 hybrid.

## 4 DISCUSSION

In this study, several banana varieties were identified as resistant to *P*. *fijiensis*, with a reaction like that of Calcutta 4. These varieties include Long Tavoy, Pahang, Malaccensis 0074, Pisang KRA, M.A. Truncata, Balbisiana, and Tani that are diploids and can be useful in breeding programmes. *P*. *fijiensis* resistance has been reported before in wild diploids like Krasan Saichon, Zebrina, Birmanie, and Tuu Gia, while others were moderately resistant (Nascimento et al., 2020). Here, most of the accessions (25 out of 31) that were resistant or had an intermediate response to *P*. *fijiensis* were diploid bananas. The resistant diploids belonged to *M*. *acuminata* subsp. *burmannica*, subsp. *malaccensis*, and subsp. *zebrina*, and to *M*. *balbisiana*. Earlier studies of banana varieties in Cameroon also reported resistant accessions in these subgroups (Fouré, 1994). More accessions from these subspecies should be screened to expand the available sources of resistance to *P*. *fijiensis*. Improved diploids are routinely used as male parents in banana breeding programmes (Swennen & Vuylsteke, 1993; Vuylsteke et al., 1993). The improved diploids 10969S‐1 and TMB2X5265‐1, for instance, were reported as good sources of black Sigatoka resistance (Batte et al., 2019). However, in the current study they were not resistant to *P*. *fijiensis*. These contrasting observations could be attributed to the high genetic diversity and emergence of new and highly virulent pathotypes arising from frequent sexual reproduction documented in *P*. *fijiensis* isolates from Uganda (Kimunye et al., 2021). Additional studies to characterize the virulence of *P*. *fijiensis* population are recommended.

Symptom progression in Calcutta 4 and other accessions in this study stopped at the early streak stage (Stage 2). This corresponds to the host reaction previously described by Meredith and Lawrence (1970) and Fouré (1994). The reaction in Calcutta 4 has been described as a hypersensitive response (Fouré, 1994; Guzmán et al., 2019), a type of resistance thought to be controlled by a major gene. This kind of resistance is readily transferable from one genotype to another but can easily be overcome by the pathogen (McDonald & Linde, 2002). Thus, as a proactive measure, other sources of resistance should be explored and incorporated into *Musa* breeding programmes.

Symptom progression in other resistant varieties such as Pisang Lilin (ITC 1121) and Monyet (ITC 1179) stopped at Stage 3, and at Stage 4 in Cacambou (ITC 0058). Although these accessions grouped with Calcutta 4, their reaction is more appropriately described as intermediate or partial resistance comparable to reaction Type 2, characterized by typical but slow symptom progression up to necrosis (Fouré, 1994). This is because they allowed the pathogen to produce asexual spores, which are normally produced at Stage 3 of symptom development (Meredith & Lawrence, 1970), despite having a higher YLS and INSL. Partial resistance suggests involvement of multiple resistance alleles or genes; thus, it is different from those accessions where symptom development stopped at Stage 2. Resistance derived from these accessions is more desirable and could be exploited in stabilizing resistance to *P*. *fijiensis*; however, it is difficult to handle/manipulate in a breeding programme. Accessions identified in this study can be included in the breeding programmes to provide breeders with an expanded genetic pool of resistance genes and avoid the overreliance of resistance genes from Calcutta 4.

Based on the AUDPC, the response of 31 accessions to *P*. *fijiensis* did not differ from the resistant check, Calcutta 4. Some of these accessions with a low AUDPC developed symptoms that progressed to Stage 6. These accessions included Duningi (ITC 0947), Bagul (ITC 0814), Buitenzorg (ITC 1178), and Kokopo (ITC 1243). This means that, although disease severity was curtailed, the pathogen continued to develop to sporulation. Some accessions that clustered with Calcutta 4 had a significantly higher AUDPC, suggesting that the use of AUDPC alone can lead to an inconsistent assessment of resistance in banana. It is therefore important to combine the AUDPC with the most advanced stage of symptom development (stage at which symptom progression stops) when screening banana accessions for resistance to *P*. *fijiensis*, even though YLS and INSL are still commonly used by researchers. The AUDPC and most advanced symptoms had a higher coefficient of determination and correlated well with YLS and INSL. Establishing how far an accession allows *P*. *fijiensis* symptom development (i.e., stage at which symptom progression stops) has not been used before and presents a fast and reliable selection criterion, especially when selecting potential breeding materials.

A substantial number of accessions resistant to black Sigatoka contained the B genome (BB, AB, AAB, ABB). This is contrary to the findings of Fouré (1994), who reported that cultivars with a B genome were mainly susceptible or had partial resistance. *M*. *balbisiana* has several desirable attributes including drought tolerance (Ravi et al., 2013), but their inclusion in banana breeding has been limited until now, primarily due to the banana streak virus (eBSV) that is encoded in the B genome (Bakry et al., 2009). Recent studies have shown that the recombination of *M*. *balbisiana* and *M*. *acuminata* resulted in an eBSV‐free progeny (Noumbissié et al., 2016; Umber et al., 2016). This presents the possibility of using *M*. *balbisiana* to broaden and improve resistance to black Sigatoka, as well as to introduce other desirable traits such as drought tolerance.

In this study, tetraploid hybrids derived from the cross of the *P*. *fijiensis*‐susceptible EAHBs (Nante, Nfuka, and Entukura) with the *P*. *fijiensis*‐resistant Calcutta 4 were susceptible to black Sigatoka. These included 376K‐1 (Nante × Calcutta 4), 222K‐1 (Nfuka × Calcutta 4), and 1438K‐1 (Entukura × Calcutta 4). The tetraploids were derived from genetically related Matooke bananas (Němečková et al., 2018) that are highly susceptible to black Sigatoka. This deviates from earlier findings whereby susceptible plantain triploids were crossed with diploid Calcutta 4, resulting in mostly black Sigatoka‐resistant tetraploid hybrids (Vuylsteke et al., 1993). Thus, selections for advancement in breeding need to be made based on the reaction of individual hybrids to black Sigatoka. Resistance to *P*. *fijiensis* in *Musa* hybrids is conferred by a major recessive gene *bs1* and two modifiers genes, *bsr_1_* and *bsr_2_*, with an additive effect (Craenen & Ortiz, 1997; Ortiz & Vuylsteke, 1994). Segregation of the three loci result in progeny with a variable response to *P*. *fijiensis* (Ortiz & Vuylsteke, 1994), thus making progeny predictions based on parental phenotype unreliable. An understanding of the genetics of resistance of a parental cultivar can guide breeders to make informed decisions on the choice of parents to use in their breeding programmes, to minimize the risk of a breakdown in resistance.

The accessions Saba, IC2, and Pelipita, which were susceptible to *P*. *fijiensis* in the current study, were previously reported as moderately resistant in Cameroon (Fouré, 1994; Guzmán et al., 2019). Pisang Ceylan was reported resistant in this study, but moderately resistant in Cameroon (Guzmán et al., 2019). These results are probably a reflection of differences in environmental factors, including different weather patterns, soil characteristics, and fertility regimes, and/or the presence of isolates differing in virulence profiles. It is therefore important that environmental factors and pathogen profiles be investigated at different locations to understand what other factors influence genotype response to infection.

This study identified several banana accessions resistant to *P*. *fijiensis*, in addition to Calcutta 4, which could be used for developing black Sigatoka‐resistant banana. These accessions include Long Tavoy, M.A. Truncata, Pisang KRA, Malaccensis 0074, Pahang, Balbisiana, and Tani (BB). Other potential *P*. *fijiensis* resistance sources are Borneo, Pisang Serun, Tuu Gia, Monyet, and Cacambou. However, resistance provided by these accessions needs to be stacked to develop cultivars with durable black Sigatoka resistance. This study has also revealed that the most advanced stage of symptom development, together with AUDPC, are good parameters for selecting potential sources of resistance. An understanding of the genetics of resistance is required to allow breeders to make informed decisions on which genes to use or stack to enhance the durability of black Sigatoka resistance in banana.

## CONFLICT OF INTEREST

The authors declare that there are no conflicts of interest.

## Data Availability

Data are available from the authors upon reasonable request.
